# Foot kinematics in walking on a level surface and on stairs in patients with hallux rigidus before and after cheilectomy

**DOI:** 10.1186/1757-1146-7-13

**Published:** 2014-02-13

**Authors:** Benita Kuni, Sebastian Immanuel Wolf, Felix Zeifang, Marc Thomsen

**Affiliations:** 1 Clinic for Orthopedics and Trauma Surgery, Department of Orthopedics, Trauma Surgery and Spinal Cord Injury, Heidelberg University Hospital, Schlierbacher Landstr. 200a, 69118 Heidelberg, Germany; 2 DRK-Klinik Baden-Baden, Lilienmattstraße 5, Baden-Baden 76530, Germany

**Keywords:** Cheilectomy, Hallux rigidus, Multi-segment foot model, Climbing stairs

## Abstract

**Background:**

Walking down stairs is a clinically relevant daily activity for older persons. The aim of this pilot study was to investigate the impact of cheilectomy on walking on level ground and on stairs.

**Methods:**

3D motion analysis of foot kinematics was performed in eight patients with hallux rigidus and 11 healthy control participants with a 12-camera system, using the Heidelberg foot measurement method before and one year after surgery. The clinical results were documented using the AOFAS Scale.

**Results:**

The range of motion of the first metatarsophalangeal joint did not improve after the operation under any gait condition. Preoperatively, hallux dorsi-/plantarflexion in level walking was 11.9° lower in patients than in controls (*p* = 0.006), postoperatively 14.5° lower (*p* = 0.004). Comparing walking conditions in patients, hallux dorsi-/plantarflexion was significantly higher in level walking than in climbing stairs (difference up stairs – level: -8.1°, *p* = 0.018).

The AOFAS Scale improved significantly from 56.9 ± 19.9 points (mean ± SD), preoperatively, to 75.9 ± 13.9 points, postoperatively (*p* = 0.027).

**Conclusions:**

Cheilectomy is appropriate for reducing symptoms of hallux rigidus. However, neither a positive influence on the range of motion in walking on level ground and on stairs nor a functional improvement was observed in this group of patients.

**Trial registration:**

NCT01804491

## Background

Cheilectomy is presently the standard operative procedure performed to treat "hallux rigidus" if the loss of articular cartilage is limited to the dorsal parts of the joint and pain persists after conservative treatment [1]. In this procedure, the dorsal third of the articular surface of both joint partners is removed. According to the literature [2,3], this procedure shows an excellent outcome with respect to passive ROM, patient satisfaction, and pain reduction [4]. Assessing passive ROM is the most common clinical test for evaluating the outcome of this type of operation. However, ROM tested passively does not correlate well with ROM tested under conditions of load; active ROM during weight bearing and heel rise correlates better with the motion of the MTP-I joint during gait [5].

Therefore, evidence concerning the efficacy of cheilectomy is poor [6]. Only few studies have shown functional improvements following this procedure: Nawoczenski et al. [7] found a significantly higher dorsiflexion and hallux abduction using electromagnetic tracking. Despite clinical improvements in pain and passive ROM, however, similar improvements in ROM were not demonstrated during ambulatory testing by means of the Milwaukee Foot Model [8].

The reason for the discrepancy between clinical and motion analysis results might be that level walking was not challenging enough to demonstrate any postoperative changes. Hence, we hypothesized that improvements in ROM may become evident when walking on stairs as patients with hallux rigidus have reported problems especially when walking down stairs.

Specifically, we suspected that lowering the body to the next step would potentially require a greater ROM of the MTP-I joint and ankle as has previously been shown for hip and knee ROM in climbing stairs [9-11].

Therefore, the aim of this pilot study was to investigate the kinematic characteristics of multi-segmental foot motion in patients with hallux rigidus before and after cheilectomy both when walking on level ground as well as on stairs. We hypothesized that the ROM of the MTP-I joint would be lower than for healthy control participants and that, after cheilectomy, the ROM would improve towards normal values, especially when walking up and down stairs. Walking speed might improve after normal kinematics are restored and pain is reduced.

## Methods

The patients were recruited from the outpatient department (foot and ankle) at our institution. All consecutive patients with an indication for cheilectomy but who did not present criteria for exclusion were included in the study. Exclusion criteria were previous operations on the foot and ankle, rheumatic diseases, and relevant foot deformities other than hallux rigidus. Fourteen consecutive patients with the indication for cheilectomy were initially included. Eight patients participated in both test sessions. In all cases the reason for refusal/drop out, unfortunately, was the long duration of the measurement. Eight patients (59.1 ± 6.4 years, mean ± standard deviation (SD), BMI 26.2 ± 2.5 kg/m^2^, six women, two men) could be tested on the day prior to the operation and at 1.1 ± 0.3 years after the operation between 10/2006 and 10/2008.

In our department, cheilectomy is performed in patients presenting with a hallux rigidus grade I or II [12] who are suffering from painful osteophytes (footwear) and pain in passive joint motion (at extremes of the joint excursion) and in whom conservative treatment did not provide pain relief. Up to grade II, the joint space narrowing is mild-to-moderate, and no more than a fourth of the dorsal joint space is involved [12]. In cases of more severe osteoarthritis in the MTP-I joint (higher grade than II), we mostly perform a fusion procedure. In seven patients surgery was performed unilaterally and in one additional case bilaterally. Medical history and present conditions regarding pain, activity level, and footwear were obtained using interviews and the American Orthopaedic Foot and Ankle Society (AOFAS) Hallux Metatarsophalangeal-Interphalangeal Scale [13]. In this scale, the hallux ROM limitation is graded as mild (higher or equal to 75% of the full ROM), moderate (30 to 74%), or severe (under 30%).

At the time of postoperative testing a stable ambulatory pattern (without any major subjective and visual quality change over the last three months) had been achieved in all cases.

All patients were operated by using a medial approach and with the same operative procedure, removing the dorsal third of both joint components of the MTP-I joint. The capsular component was closed loosely in order to allow a maximum ROM postoperatively. Full weight bearing and normal footwear were allowed directly after the operation. The patients were instructed to exercise the maximum excursion of the MTP-I joint as soon as possible and on a regular basis.

As a control group, 11 healthy participants (mean age: 50.2 ± 8.6 years, BMI 23.0 ± 3.9 kg/m^2^, seven women, four men) without any foot deformity, previous foot operations, or pain at the lower extremity were recruited from the local population and tested between 09/2007 and 09/2008. All participants gave their written, informed consent. The procedures and the test protocol were approved by the Ethics Committee of the Medical Department of the University and followed the World Medical Association Declaration of Helsinki.

### Data acquisition

**Figure 1:**
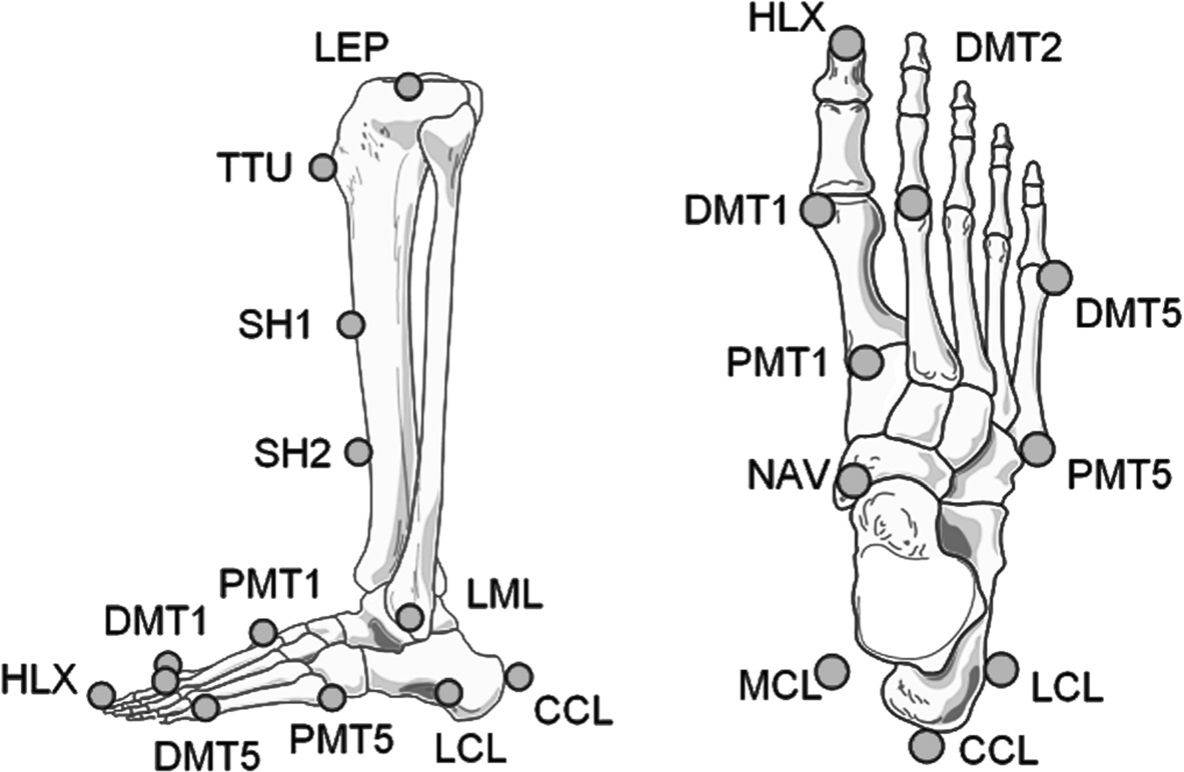
Marker placement according to the Heidelberg Foot Measurement Method*. Hallux (HLX), metatarsal heads (DMT1, DMT2 and DMT5), proximally at the 1st and 5th metatarsal (PMT1, PMT5), navicular (NAV), lateral malleolus (LML), dorsal (CCL), medial and lateral (MCL, LCL) calcaneus, lateral epicondyle (LEP), tibial tuberosity (TTU), shin (SH1/2) markers; the medial epicondyle (MEP) and medial malleolus (MML) markers are not shown. *Reprinted from Gait & Posture, 23 (4), J. Simon, L Doederlein, A.S. McIntosh, D. Metaxiotis, H.G. Bock, S.I. Wolf, The Heidelberg foot measurement method: Development, description and assessment, Page 414, 2006, with permission from Elsevier.

All trials were performed barefoot using the marker setup and protocol according to the Heidelberg foot measurement method (HFMM) [14]. Seventeen retro-reflective markers 6 mm in diameter were attached to the skin on each leg (Figure 1), namely, on the distal phalanx of the hallux (HLX), the metatarsal heads (DMT1, DMT2, and DMT5), proximally at the 1st and 5th metatarsal (PMT1 and PMT5), the navicular (NAV), the medial and lateral malleolus (MML and LML), and the dorsal position of the calcaneus (CCL), each placed with the participant in a standing posture. The medial and lateral heel markers (MCL, LCL) were placed with the aid of an alignment device while the participant was sitting and the foot was not bearing any load. Five markers were placed at the tibia (LEP, MEP: lat./med. epicondyle, TTU: tibial tuberosity, SH1/2: two points on the medial side of the shin).

Marker trajectories were captured at 120 Hz with a 12-camera system (Vicon 612, Oxford-Metrics). The optical accuracy given by the residuum of the marker reconstruction algorithm was between 1 and 2 mm [14].

A static reference measurement was performed in a standing posture before the participant was asked to walk along a 7-m path at a self-selected speed. Data acquisition was repeated until eight full strides had been captured for each leg.

At least six stair ascents and descents were monitored on a custom-made 80-cm-wide staircase which consisted of five steps of 15 cm in height and a step distance of 32 cm.

### Data processing

For data processing, we only included one foot of the one patient who was operated on both feet and only one foot of each control participant (randomly chosen).

Intersegment and joint angles were calculated with the custom-made software "MoMo" within Matlab (v6.5.1) following the method described by Simon et al. [14]. This software also served to normalize the time data to the gait cycle, to average trials for each participant, to visualize charts, and to make temporospatial calculations. For further data analysis motion data were not filtered in any way since after averaging across at least 8 trials (6 for stairs) only little higher frequency content was found. Unlike typical models, which assume a series of two or three rigid and rather artificial segments in the foot and allow artificial joints of typically 3 degrees of motion (rotations) as represented by Euler- or Euler-Cardan angles, the HFMM describes the angular orientations of anatomical landmarks, possibly spanning more than one anatomical joint, rather than relying solely on rigid segment modeling. Such a "functional segment" is then described by its relative motion via projection angles defined as the angle between two vectors (or 2D-segments) in the perspective view along the axis of rotation. Consequently, the motion of the ankle complex is described by two axes of rotation accounting for talocrural and subtalar motion via the motion of the three calcaneal markers (CCL, LCL, MCL) and the navicular marker (NAV) with respect to tibial markers (LEP, TTU, SH1, SH2). For ease of interpretation, the medial arch is defined directly as the angle spanned by the triangle of the markers MCL, NAV, and PMT1. For further details concerning the model, we refer the reader to Simon et al. [14]. The reliability (between stride, rater, and day) of the HFMM parameters has already been tested in a previous study [14]. The reliability values for the hallux dorsi-/plantarflexion ROM were mean standard deviations (SD) and coefficients of multiple correlation (CMC) stride-to-stride: SD 1.37, CMC 0.993; day-to-day: SD 1.97, CMC 0.984; and inter-rater: SD 2.80, CMC 0.970. According to Simon et al. [14] the HFMM parameters show standard deviations between two and seven degrees for absolute angular values due to inaccurate marker placement on the part of the examiner but standard deviations in ROM remain small, at between 0.3° and 1.8°.

The ROM for the following parameters was chosen in order to describe the gait kinematics in the three different walking conditions (level, up stairs, and down stairs): hallux dorsi-/plantarflexion, hallux ab-/adduction, medial (longitudinal) arch (Medial arch in Table 1), subtalar in-/eversion (Subtalar motion), medial/lateral tilt of the medial arch (Medial arch inclination), midfoot-ankle pro-/supination, fore-midfoot pro-/supination, fore-hindfoot ab-/adduction, the absolute angle between metatarsal I and V, projected into the transverse plane (Metatarsal I-V angle), dorsi-/plantarflexion in the tibio-talar joint (Talocrural motion (HFMM) in Table 1) [14], and conventional ankle motion [15]. The latter parameter describes the motion of the complete foot, including mid- and forefoot motion relative to the tibia simply as a "heel" and a "toe" marker, whereas "Talocrural motion (HFMM)" [14] explicitly describes talocrural motion of the hindfoot, which is referred to as "ankle dorsi-/plantarflexion" or "ankle joint range of motion" in this paper.

**Table 1:** Intersegmental foot range of motion (in degrees) for the control group (Contr.) and the patients (Pat.) before (preop.) and after (postop.) the operation for all gait conditions (level, up and down stairs)*

	**Contr.**		**Pat. preop.**		**Pat. postop.**		**Pat. preop. vs. contr.**			**Pat. postop. vs. contr.**			**Pat. postop. vs. preop.**		
**Level**	**Mean**	**SD**	**Mean**	**SD**	**Mean**	**SD**	***P-*** **value**	**Cohen’s d**	**Effect size r**	***P*-value**	**Cohen’s d**	**Effect size r**	***P*-value**	**Cohen’s d**	**Effect size r**
Hallux dorsi-/plantarflexion	**49.3**	6.4	**37.4**	8.3	**34.8**	9.7	**0.006**	-1.61	-0.63	**0.004**	-1.76	-0.66	**0.036**	-0.29	-0.14
Hallux ab-/adduction	**6.4**	1.8	**3.9**	1.4	**4.4**	1.6	**0.005**	-1.55	-0.61	**0.017**	-1.17	-0.51	0.674		
Talocrural motion (HFMM)	**22.5**	5.3	**16.7**	3.1	**17.5**	3.4	**0.026**	-1.34	-0.56	**0.026**	-1.23	-0.49	0.327		
Ankle motion (conventional)	**33.5**	6.5	**24.8**	6.6	**24.8**	6.0	**0.013**	-1.33	-0.55	**0.013**	-1.39	-0.57	0.575		
Subtalar motion	**10.2**	2.6	**8.7**	2.4	**9.8**	4.1	0.215			0.804			0.327		
Medial arch (cavus/planus)	**18.3**	2.6	**13.8**	5.6	**14.1**	5.9	0.099			0.090			0.735		
Medial arch inclination (med./lat.)	**15.1**	4.4	**14.3**	5.6	**14.8**	6.7	0.934			0.869			0.779		
Midfoot-ankle pro-/supination	**12.2**	2.6	**11.4**	3.9	**13.4**	6.2	0.563			0.741			0.123		
Fore-midfoot pro-/supination	**6.8**	2.0	**4.4**	2.0	**6.3**	3.0	**0.032**	-1.20	-0.52	0.248			**0.025**	0.75	0.35
Fore-hindfoot ab-/adduction	**8.9**	2.2	**6.2**	3.0	**6.3**	2.3	**0.048**	-1.03	-0.46	**0.039**	-1.16	-0.50	0.484		
Metatarsal I-V angle	**10.7**	1.9	**10.1**	2.1	**10.5**	2.2	0.409			0.509			0.484		
**Up stairs**															
Hallux dorsi-/plantarflexion	**33.6**	7.0	**27.7**	7.8	**27.3**	7.8	0.189			0.113			0.735		
Hallux ab-/adduction	**6.3**	2.4	**8.2**	4.8	**4.5**	1.4	0.497			0.113			0.063		
Talocrural motion (HFMM)	**27.9**	3.6	**26.8**	5.7	**26.9**	6.6	0.684			0.892			0.866		
Ankle motion (conventional)	**37.9**	4.9	**33.8**	7.3	**35.1**	8.8	0.221			0.497			0.310		
Subtalar motion	**10.7**	2.3	**9.7**	3.3	**10.8**	4.3	0.618			0.964			0.499		
Medial arch (cavus/planus)	**15.8**	3.5	**12.8**	4.3	**13.0**	4.8	0.077			0.258			0.866		
Medial arch inclination (med./lat.)	**13.5**	4.3	**15.8**	4.0	**12.8**	4.9	0.221			0.821			0.091		
Midfoot-ankle pro-/supination	**11.5**	3.4	**11.2**	3.6	**12.9**	6.9	0.751			0.821			0.499		
Fore-midfoot pro-/supination	**6.3**	1.6	**4.2**	1.9	**5.1**	2.8	**0.016**	-1.20	-0.51	0.160			0.176		
Fore-hindfoot ab-/adduction	**6.4**	2.5	**5.5**	2.3	**6.0**	3.4	0.441			0.821			0.310		
Metatarsal I-V angle	**11.2**	2.4	**12.4**	3.5	**11.4**	1.5	0.497			0.751			0.398		
**Down stairs**															
Hallux dorsi-/plantarflexion	**39.5**	8.3	**31.3**	4.0	**29.7**	8.6	**0.021**	-1.26	-0.53	**0.026**	1.16	0.50	0.866		
Hallux ab-/adduction	**7.4**	2.7	**5.1**	1.4	**4.2**	1.6	0.094			**0.008**	1.44	0.59	0.398		
Talocrural motion (HFMM)	**46.5**	4.8	**45.8**	7.1	**44.9**	5.8	0.618			0.258			1.000		
Ankle motion (conventional)	**61.2**	5.5	**55.4**	7.1	**55.9**	5.9	0.077			0.052			1.000		
Subtalar motion	**11.6**	3.7	**10.9**	2.6	**9.3**	2.9	0.684			0.221			0.063		
Medial arch (cavus/planus)	**21.3**	3.4	**17.1**	4.4	**18.2**	2.9	0.077			0.113			0.398		
Medial arch inclination (med./lat.)	**15.4**	5.0	**15.4**	5.2	**13.4**	5.6	0.964			0.390			**0.043**	-0.37	-0.18
Midfoot-ankle pro-/supination	**13.6**	3.9	**11.6**	3.4	**12.9**	3.6	0.441			0.964			0.176		
Fore-midfoot pro-/supination	**7.8**	1.4	**4.8**	1.1	**5.7**	1.2	**0.002**	-2.38	-0.77	**0.010**	-1.61	-0.63	0.063		
Fore-hindfoot ab-/adduction	**5.4**	1.3	**5.7**	1.7	**4.9**	0.9	0.751			0.390			0.237		
Metatarsal I-V angle	**12.7**	3.1	**12.4**	3.0	**11.6**	2.7	0.751			0.441			0.866		

*Mean and standard deviation (SD); *p* -values are given for group comparisons and repeated measures, respectively, and marked in bold if significant on the level of alpha = 0.05.

For hallux dorsi-/plantarflexion, the ROM was also determined in seven functional subphases. In addition, the walking speed was determined in all gait conditions.

### Statistical methods

The statistical analyses were performed with SPSS 16.0.1 (SPSS Inc., Chicago, Ill, USA). Measures of central tendency and dispersion were calculated for all variables, and goodness of fit to normal distribution was assessed by using the Kolmogorov-Smirnov Test. The Wilcoxon paired-rank test was applied for pre-/post comparisons within the patient group; Mann–Whitney *U*-Test was used for group comparisons to normal references. For comparisons between conditions (level walking, stairs up and down), the Friedman test was used in order to determine overall differences. The significance level was assigned at 5% for all comparisons. Bonferroni correction was used in analyzing the seven level gait subphases. Effect sizes (Cohen’s d, effect size r) were calculated for all significant results. There is a significant difference in age (*p* = 0.032) and BMI (*p* = 0.013) between groups, which may influence the results. Therefore, regression analyses with respect to age and BMI were performed. The correlations were not significant or clinically relevant when including both patients and control participants.

## Results

### Clinical outcome

The AOFAS Scale improved significantly from 56.9 ± 19.9 points (mean ± SD) preoperatively to 75.9 ± 13.9 points postoperatively (*p* = 0.027).

Preoperatively, the pain level (AOFAS) was 18.8 ± 13.6 points (mean ± SD) and postoperatively 27.5 ± 7.1 points (0 points = severe pain, almost always present; 20 points = moderate, daily pain; 30 points = mild, occasional pain; and 40 points = no pain); according to the numbers available, no significant difference could be detected (*p* = 0.102). One patient was free of pain; four patients reported "mild" and three "moderate" pain. The activity level (AOFAS) was 5.9 ± 2.2 points preoperatively and 7.0 ± 2.7 points postoperatively (*p* = 0.083).

The preoperative limitation of the MTP-I ROM, assessed by using the AOFAS Scale, was severe in two of the eight patients, moderate in five, and mild in one. It improved to the next-better category in three cases and did not change in the others.

### Foot kinematics

The mean ROM of the foot parameters in patients and control participants in all gait conditions and the results of the comparisons between groups and between time points are shown in Table 1 (means, SD and *p-*values).

**Figure 2:**
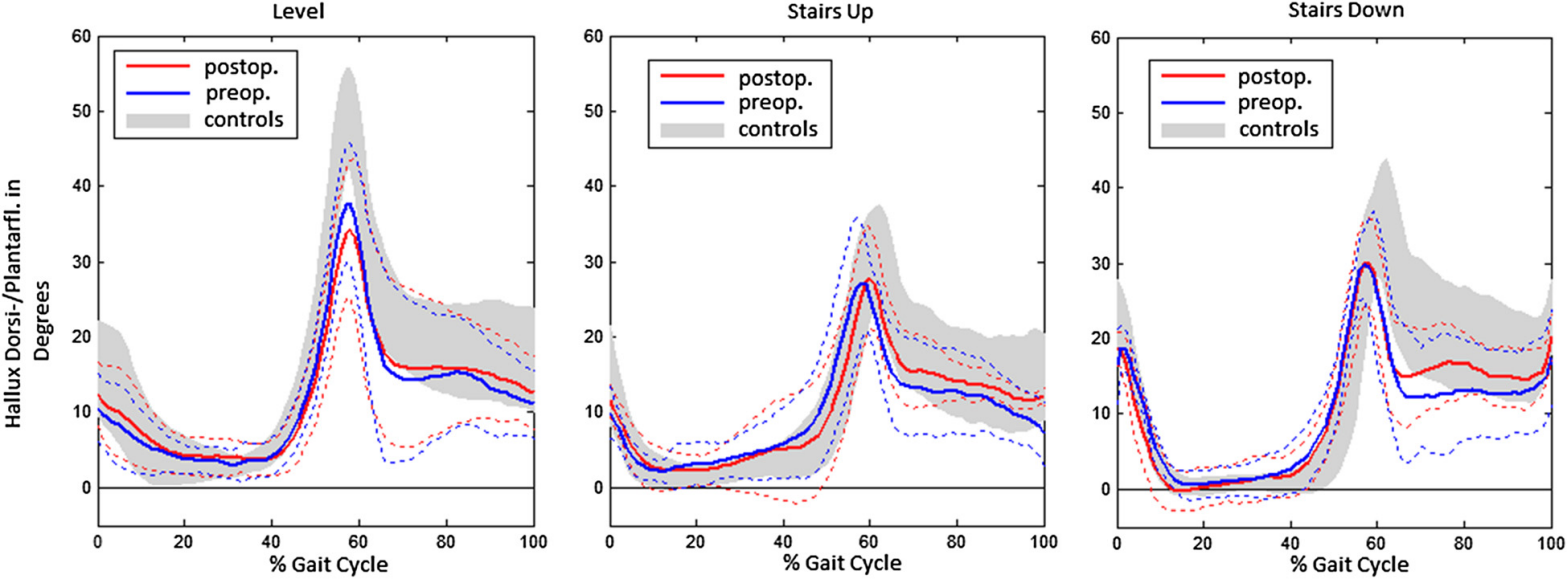
**Pre- and postoperative hallux dorsiflexion/plantarflexion.** Pre- and postoperative hallux dorsiflexion/plantarflexion given as angles (in degrees) with time of the gait cycle (0-100%) for patients compared to the control participants in all gait conditions. Heavy lines: mean values, dashed lines: standard deviation. Positive values indicate dorsiflexion.

The hallux dorsi-/plantarflexion ROM was significantly lower than in controls in level walking and descending stairs pre- and postoperatively. In the comparison between preoperative and postoperative state, the hallux dorsi-/plantarflexion ROM decreased by 2.5 degrees (*p* = 0.036) in level walking. Diagrams of the hallux ROM of all patients and control participants in all walking conditions are displayed in Figure 2.

The analysis of level gait subphases only showed postoperatively significant differences between patients and controls for the maximum hallux dorsiflexion in preswing (controls: 38.2 ± 5.8 degrees; patients preoperatively: 29.6 ± 6.0 degrees; *p* = 0.013, non-significant. after Bonferroni correction with the adjusted alpha: 0.007, postoperatively: 26.6 ± 7.1, *p* = 0.004). The preswing dorsiflexion did not improve after the operation (*p* = 0.025, non-significant after Bonferroni correction with the adjusted alpha: 0.007).

In comparison to the controls the patients showed a significantly lower ankle ROM (conventional and talocrural HFMM) pre- and postoperatively in level walking and preoperatively a reduced fore-midfoot pro-/supination in all gait conditions. In level gait, the fore-midfoot pro-/supination increased significantly between the two measurements (*p* = 0.025).

Comparing walking conditions in patients, the hallux dorsi-/plantarflexion was significantly higher in level walking than in climbing stairs (*p* = 0.018).

It was higher for walking down stairs than up stairs: in the control group (*p* = 0.033) and in the patients postoperatively (*p* = 0.043).

The talocrural motion (HFMM) was the highest when walking down stairs, followed by climbing stairs, both significantly higher than in level walking (both *p* = 0.018).

In the control group, the talocrural motion was also higher for walking stairs than for level walking (up: *p* = 0.013 and down: *p* = 0.003 versus level walking).

In both groups, the talocrural motion was higher for walking down stairs than up stairs (controls: *p* = 0.003, patients: *p* = 0.018).

### Walking speeds

Pre- and postoperative walking speeds matched in the patient group in level walking and in walking up the stairs (Table 2). Postoperatively, patients reduced their speed when walking down the stairs as compared to the preoperative speed (*p* = 0.043, Wilcoxon).

**Table 2:** Walking speeds in m/s in all gait conditions, for the control group (Contr.) and for patients (Pat.) pre- and postoperatively*

**Speed**	**Contr.**		**Pat. preop.**		**Pat. postop.**		**Pat. preop. vs. contr.**			**Pat. postop. vs. contr.**			**Pat. postop. vs. preop.**		
	**Mean**	**SD**	**Mean**	**SD**	**Mean**	**SD**	***P-value***	**Cohens’s d**	**Effect size r**	***P-value***	**Cohens’s d**	**Effect size r**	***P-value***	**Cohens’s d**	**Effect size r**
Level	**1.38**	0.15	**1.18**	0.07	**1.20**	0.12	**0.034**	1.71	0.65	**0.021**	1.33	0.55	0.484		
Up stairs	**0.52**	0.06	**0.50**	0.05	**0.49**	0.05	0.329			0.242			0.176		
Down stairs	**0.55**	0.05	**0.53**	0.05	**0.48**	0.05	0.221			**0.004**	1.4	0.57	**0.043**	-1.0	-0.45

*Mean and standard deviation (SD); *p* -values are given for Mann-Whitney U test (patients vs. controls) and Wilcoxon test (postoperatively (postop.) vs. preoperatively (preop.)), and marked in bold if significant on the level of alpha = 0.05.

For patients, walking speeds were lower than for controls in level walking (Mann–Whitney U, preoperatively *p* = 0.034, postoperatively *p* = 0.021) and walking down the stairs postoperatively (*p* = 0.004; preoperatively *p* = 0.221), but not climbing up the stairs (preoperatively *p* = 0.329, postoperatively *p* = 0.242).

## Discussion

By using the multi-segmental HFMM, segmental changes in the fore- and midfoot can be analyzed. According to Simon et al. [14], repeatability was much better for hallux motion and for most mid- and forefoot parameters than the more frequently used Oxford foot model [16]. Parameters related to the medial arch are uniquely defined in the HFFM and, to our knowledge, there is no counterpart in other model approaches. However, hallux motion as well as most mid- and forefoot parameters resemble the joint motion patterns of other approaches, for example, Leardini et al. [17], MacWilliams et al. [18], or Carson et al. [16].

In this pilot study, only few kinematic differences were found between patients presenting with a hallux rigidus grade I or II [12] and controls. The low-grade hallux rigidus did affect level walking by limiting the hallux and the ankle ROM and also the walking speed. When descending stairs, the hallux pathology could also be seen.

Hallux rigidus did not affect climbing up stairs, however. In all gait conditions, the fore-midfoot pro-/supination was reduced.

### Level walking

Cheilectomy did not restore normal hallux or ankle joint ROM in the patients of this pilot study.

The study of Nawoczenski et al. [7] found an average of 12 degrees motion recovery following cheilectomy. These authors used the proximal phalanx of the hallux instead of the distal phalanx and Cardan/Euler angle. Others [8] did not observe any significant improvements in ROM. After surgery, hallux dorsiflexion remained reduced in loading response and initial swing in those patients, but it improved during the rest of the gait phases. In our patient population, we found smaller hallux dorsiflexion in the preswing phase of gait postoperatively, indicating a slight deterioration rather than an improvement. These patients might try to secure the more mobile MTP-I joint in order to prevent pain.

Reasons other than bony abutment seem to account for the residual limitation in hallux mobility. This finding could be related to capsular shrinkage or scar tissue in case of delayed physiotherapy or mobilization after the operation. Other reasons for the small functional effects observed for cheilectomy could be due to concomitant pathological conditions such as stenosing tenosynovitis of the flexor hallucis longus, which were not assessed by the bony resection without a plantar soft tissue release. According to the graphs for the hallux ROM over the gait cycle (Figure 2), the dorsiflexion curve drops steeper than in the control group in the initial swing phase. This effect could be due to a passive force, perhaps caused by a soft tissue restriction acting as a reset force. Therefore, we strongly recommend that exercising the MTP-I ROM is begun immediately in postoperative treatment. The motion pattern of a person who has walked with a limited ROM of the MTP-I joint for years might also remain the same even one year after the operation.

Furthermore, ROM was not completely restored in the horizontal plane by the operation: The hallux ab-/adduction increased after surgery, but it was still significantly lower than in controls. The osteophytes might not have been the main cause of this restriction, but rather the morphologic changes in the MTP-I joint due to osteoarthritis. However, the restricted fore-hindfoot ab-/adduction might also be a secondary consequence of the abnormal forefoot kinematics as it did not improve during level walking after the operation. Indeed, the reduced mobility in the sagittal plane seems to cause secondary restrictions in the other degrees of freedom (ab-/adduction and pro-/supination). Comparable restrictions in the mobility of adjacent foot segments in planes (parameters: tilt of the medial arch, in-/eversion) other than the initially mostly restricted one (plantar-/dorsiflexion) were previously observed in patients with ankle osteoarthritis after joint replacement [19].

### Stairs

The preoperative limitations in the MTP-I joint were expected to be more relevant for climbing/descending stairs than for ground level gait. Contrary to our expectations, however, the hallux ROM was the highest in level walking, followed by descending stairs. Apparently, there is no need for extended ROM of the MTP-I joint while climbing stairs; compared to level walking a significantly lower ROM was found in climbing stairs.

In previous studies, the kinematics of climbing stairs showed increased knee and hip flexion in the sagittal plane as compared to level walking in healthy participants [9-11]. In addition, the ankle joint is more involved in the swing phase [20]. Greater ankle angles were found while descending stairs [21].

In both of our groups, the ankle joint ROM was significantly higher in walking stairs than in level walking. The higher need of mobility for the level change in walking stairs apparently does not involve the forefoot, but mostly the ankle joint. Our findings of higher ankle ROM while descending stairs than ascending stairs confirmed the results of previous studies [21].

The same was also found for the MTP-I joint in our healthy participants and for patients postoperatively. This finding could be interpreted to indicate a slight improvement towards a normal gait pattern.

In all gait conditions, we found a significantly lower fore-midfoot pro-/supination ROM in patients than in controls, with an improvement in level gait after the operation. Our results suggest that this effect was successfully treated by cheilectomy, although the ROM of the MTP-I joint did not change significantly. Postoperatively, the group difference only remained significant for descending stairs.

The reduced fore-/midfoot pro-/supination could represent one of the main adaptation mechanisms in hallux rigidus patients due to the limitation in the MTP-I joint. As rolling over the first ray is limited, the foot might stay in a more locked and supinated position in the mid- to forefoot area. Therefore, this ROM was also reduced as a secondary consequence of the pathology in the MTP-I joint. The difficulty hallux rigidus patients have in walking down steps could also be due to the additional rigidity in the midfoot. The operation might have had an additional negative influence on the sense of security for walking down stairs as patients also reduced their walking speed.

### Clinical outcome

The main goal of any operative intervention to treat hallux rigidus is to reduce pain. Although there was no statistical evidence of pain relief, we found an overall improvement in the total AOFAS Scale after the operation, indicating a better clinical state. The lack of significant changes in the subcategories pain and activity could be due to the small number of patients. The validity of the AOFAS Hallux Metatarsophalangeal-Interphalangeal Scale has previously been shown to be questionable, especially related to activity [22]. Not all the subcategories are useful for specifying the pathology of hallux rigidus: Since ROM is not differentiated in dorsiflexion and plantarflexion of the MTP-I joint, restrictions and changes in the MTP-I joint dorsiflexion are not always well represented. The motion of the interphalangeal joint of the hallux and the stability of the metatarsophalangeal-interphalangeal joint are mostly not impaired by hallux rigidus. Therefore, these subcategories do not reflect the changes achieved by cheilectomy, in which osteophytes are always removed. The weighting of the alignment seems to be mostly chosen with respect to hallux valgus patients. Therefore, these subcategories are not suitable for distinguishing the degree of hallux rigidus and quantifying the results of the operative procedure. However, the total scale sum seemed to reflect the state of the patient quite well since the main points are composed of subjectively reported data.

### Limitations of the study

The minor effect of the operation on foot kinematics and kinetics may be due to selecting patients with hallux rigidus grade I and II [12]. Usually, only these patients would be treated by cheilectomy; in patients with more severe disease and greater ROM limitations, arthrodesis would be performed or a hemi-prosthesis implanted. Patients with a more severe degree of osteoarthritis in the MTP-I joint would probably show greater changes in these outcome parameters after the operation.

Furthermore, the relatively low sample size needs to be mentioned. Therefore, this study should be considered as a pilot study. Prospectively, the effects of cheilectomy on hallux function in walking stairs were lower than expected. However, our data show clinically relevant differences between the pathologic and physiologic foot kinematics. Therefore, using the HFMM seems to be appropriate for investigating the pathological state of the MTP-I joint. The HFMM does not directly measure MTP-I joint motion. Rather, it measures motion of the distal phalanx with respect to the first metatarsal. However, we excluded major compensatory changes in the first interphalangeal joint (i.e., hyperextension deformities) in the thorough clinical assessment. In addition, previous studies have implicated (dorsal) translation of the first metatarsal [23-25] that may minimize angular changes of the proximal phalanx. The HFMM might miss these kinematic responses.

## Conclusions

Cheilectomy has been thought to provide higher ROM of the MTP-I joint. With respect to the results of this study, however, patients should not expect a relevant gain in hallux dorsiflexion or better function in daily movements such as walking stairs from that operation. Supplementary plantar soft tissue release might be useful in order to achieve a better ROM.

Cheilectomy improved the patients’ conditions as measured by the total sum of the AOFAS Hallux Metatarsophalangeal-Interphalangeal Scale. However, neither was a positive effect of cheilectomy on the ROM of the MTP-I joint nor functional improvement in level and stairs walking observed in this pilot study.

## Abbreviations

AOFAS: American Orthopaedic Foot and Ankle Society; MTP-I: First metatarsophalangeal joint; ROM: Range of motion; HFMM: Heidelberg foot measurement method.

## Competing interests

The study was funded by the Ministry of Science Baden-Württemberg, Germany. None of the authors has a conflict of interest.

## Authors’ contributions

BK, SW: conception and design, acquisition of data, analysis and interpretation of data, drafting the manuscript, revising the manuscript critically for important intellectual content, final approval of the manuscript version to be published. FZ: acquisition of data, drafting the manuscript, revising the manuscript critically for important intellectual content, final approval of the manuscript version to be published. MT: conception and design, acquisition of data, analysis and interpretation of data, revising the manuscript critically for important intellectual content, final approval of the manuscript version to be published. All authors read and approved the final manuscript.
